# Mobile health apps for systemic lupus erythematosus and lupus nephritis: a critical appraisal

**DOI:** 10.1186/s13075-022-02791-0

**Published:** 2022-05-14

**Authors:** Akshara Ramasamy, Poojitha Dugyala, Chandra Mohan

**Affiliations:** Biomedical Engineering Department, University of Houston, 3517 Cullen Blvd., Room 2027, Houston, TX USA

**Keywords:** Systemic lupus erythematosus, Lupus nephritis, Autoimmune diseases

## Abstract

**Objectives:**

To critically assess the quality and functionality of the available mobile apps for systemic lupus erythematosus and lupus nephritis patients.

**Methods:**

Two reviewers independently searched the App Store and Google Play Store for eligible mobile health (mHealth) apps. Two separate searches were done: one for systemic lupus erythematosus (SLE) and the other for lupus nephritis (LN). The Mobile App Rating Scale (MARS) was used to rate the quality of all selected apps.

**Results:**

From the systemic lupus erythematosus screening, our search yielded 841 apps. Within these 841 apps, 17 of them were ultimately included. From the lupus nephritis screening, our search returned 1152 apps. Of the 1152 apps, 2 were ultimately included. Our search strategy included apps specifically designed for patients with SLE and LN. The MARS average of all the systemic lupus erythematosus apps was 2.7 out of 5. The MARS average of the two lupus nephritis apps was 2.6 out of 5.

**Conclusion:**

Mobile health apps can serve as an effective tool for telehealth, engaging patients in self-care and for increasing the quality of life of lupus patients. While several mobile health technologies exist for patients with SLE and LN, there is still a significant need for app quality improvement and expanding the comprehensiveness of offered functions.

## Background

Systemic lupus erythematosus (SLE) is a chronic disorder in which the body’s immune system attacks its own tissues, causing widespread inflammation. SLE affects at least 1.5 million individuals in the USA, with 16,000 new cases recorded each year [1]. Lupus nephritis (LN) is a complication of SLE with manifestations such as kidney inflammation and eventual renal failure [2]. Approximately 40% of adults and 50–80% of adolescents with systemic lupus erythematosus will develop lupus nephritis, as early as a year within diagnosis [2–4]. Conventional treatment for LN includes high-dose corticosteroids and immunosuppressive agents [5, 6]. While pharmacologic treatments have increased patient survival overall, drug therapies are associated with a wide range of adverse effects, and corticosteroids have caused significant treatment-associated toxicity [5, 6]. In recent years, both patient progress and immunosuppressive treatment for LN have stagnated. At 12 months, short-term complete renal response rates are 10–40%, long-term outcomes have not adequately improved, and end-stage kidney disease may develop in 10–30% of individuals with LN [5, 6]. In addition, one of the most common causes of treatment failure is non-adherence to pharmaceutical therapy [7].

SLE and consequently LN have a significant impact on a patient’s quality of life, which is heavily influenced by the psychosocial components of the disease [8]. Though pharmacologic treatments address the clinical manifestations of LN, alternative therapies are needed to address the psychological symptoms such as cognitive difficulties, depression, and fatigue. Over the past decade, there has also been a growing interest in non-pharmacologic therapies as supplementary treatment options for LN patients. Psychological interventions such as psychotherapies have shown to be beneficial as adjuncts to traditional medical treatment in LN patients for improvement in fatigue, depression, pain, and quality of life [9].

With the advent of non-pharmacological treatment options, there has also been a rise of health information technology (HIT) such as disease-specific apps for mobile devices. In recent years, HIT has become increasingly important in rheumatic illnesses, particularly the care of patients with SLE and LN, for diagnostic, therapeutic, and prognostic purposes, as well as disease management [8]. Mobile phones and wireless technologies utilized in healthcare (mHealth) help bridge the gap between the patient and the healthcare practitioner by providing physicians with a realistic summary of the patient’s health state in between consultations. The more easily symptoms and changes can be captured in real time and communicated to a healthcare practitioner, the better the condition may be controlled. As SLE/LN symptoms vary greatly on a daily basis and are unique to the individual, mHealth holds promise in the management of autoimmune diseases. For example, a clinical trial by Mymee Inc., a digital health company dedicated to autoimmune disease research, demonstrated that the Mymee Digital Health Program resulted in statistically significant improvements in health-related quality of life (HRQoL) in lupus patients when combined with standard therapy [10].

In the USA, approximately 85% of Americans own smartphones [11]. This percentage increases within the young adult population, with 95% of American teenagers having access to a smartphone [12]. Due to the widespread use of smartphones, mHealth technologies play an increasingly significant role in SLE/LN management, particularly among young adults, who have more severe symptoms [1]. Understanding the quality and functionality of the available mobile health apps for lupus patients is crucial to decreasing the communication gap between the physician and patient and increasing the patient’s overall quality of life. This systematic review and critical appraisal aim to study the functionality and quality of the available mHealth apps for SLE and LN patients, using well-accepted tools and metrics. Understanding the role mHealth apps play in disease management is critical for clinicians to build patient-centered treatment plans and for the overall well-being of individuals with SLE and LN.

## Methods

**Fig. 1:**
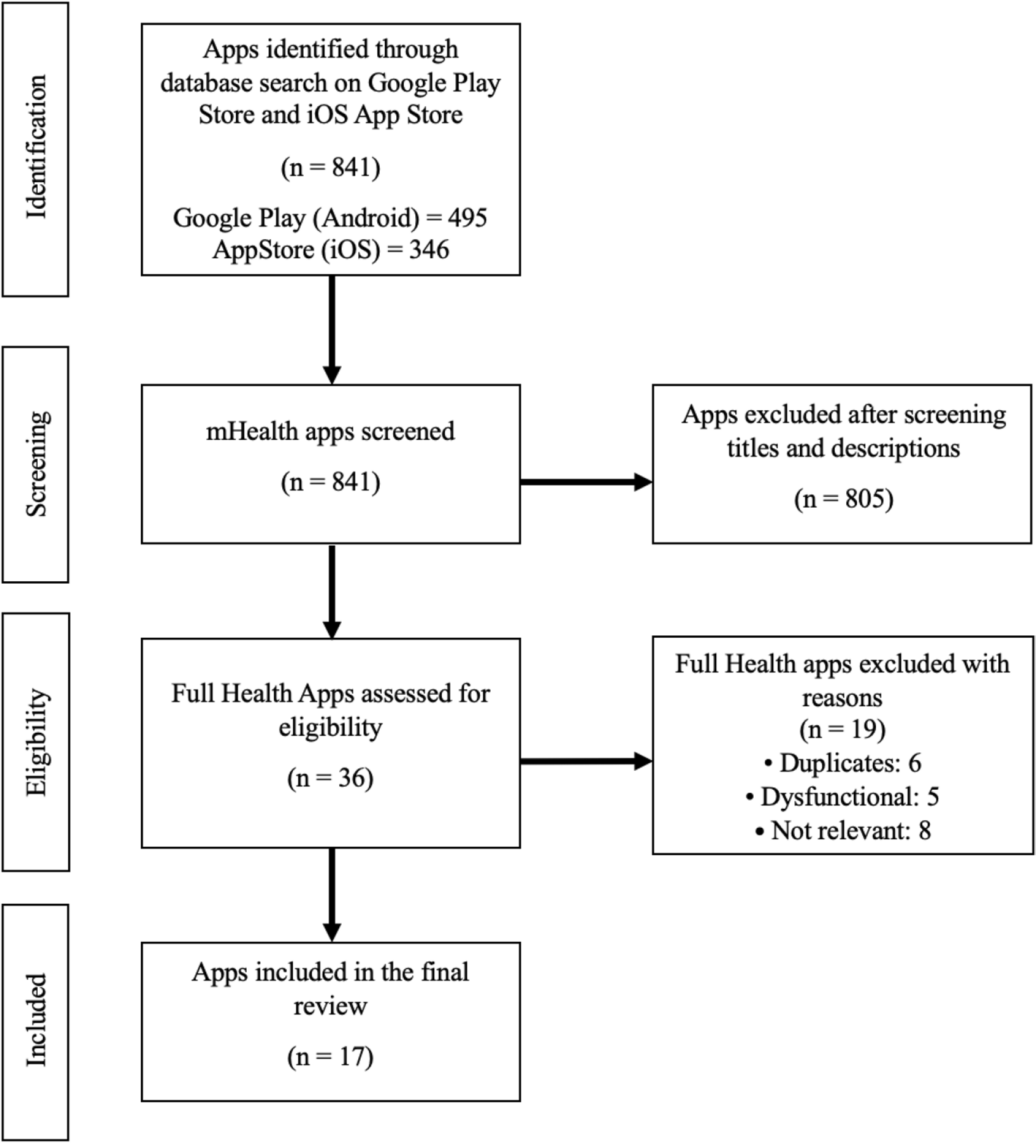
PRISMA diagram outlining all SLE apps that were initially identified and subsequently excluded or included for analysis. This figure follows the flow of the SLE app identification and selection process. The initial database search yielded 841 applications, with 495 apps from the Google Play Store and 346 from the App Store. After all 841 apps underwent a title and app description screening, 805 apps were excluded due to their irrelevance to SLE patients. This process left 36 apps eligible for a full app screening. For the following reasons, 19 applications were eliminated from the second phase of screening: there were 6 duplicates (the same app appeared in more than one search), 5 apps did not function, and 8 apps were unrelated to the purpose of this review. Following the full app screening, 17 apps were ultimately included in the final analysis

**Fig. 2:**
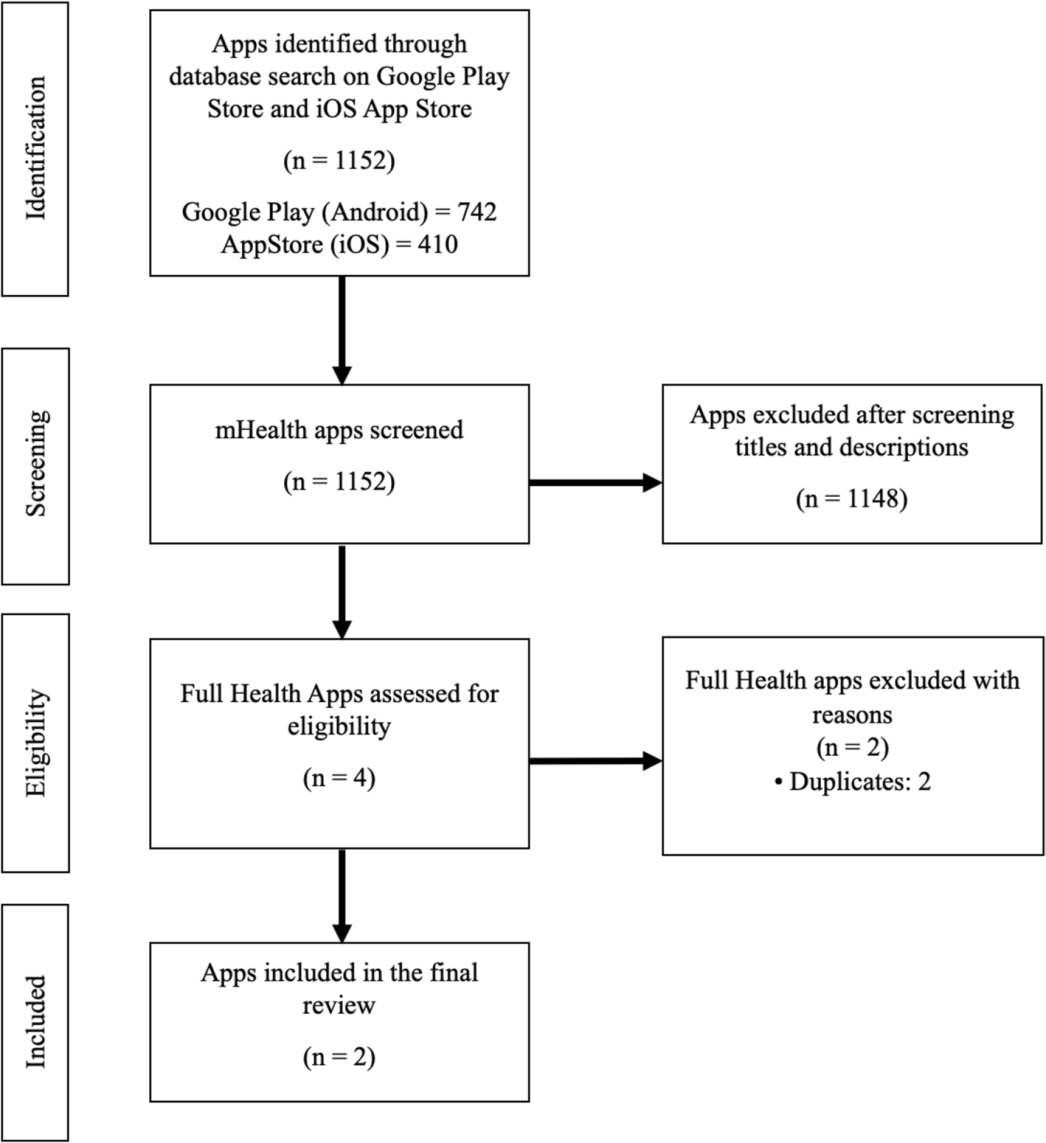
PRISMA diagram outlining all LN apps that were initially identified and subsequently excluded or included for analysis. This figure follows the flow of the LN app identification and selection process. Our initial database search yielded 1152 applications, with 742 apps from the Google Play Store and 410 from the App Store. After all 1152 apps underwent a title and app description screening, 1148 apps were excluded due to their irrelevance to LN patients and the purpose of this review. This process left 4 apps eligible for a full app screening. Due to duplicates, 2 applications were eliminated from the second phase of screening. Following the full app screening, 2 apps were ultimately included in the final analysis

Two reviewers (AR and PD) independently searched the App Store (iOS) and USA Google Play Store (Android) to screen, review, and abstract eligible mHealth apps. Our search terms for the systemic lupus erythematosus screening included lupus and SLE. For the lupus nephritis screening, our search terms included nephritis, kidney, and CKD. Originally, “kidney” and “CKD” were not part of our search strategy. However, the search term “nephritis” did not yield any apps designed for LN patients, so we increased the scope of search by adding “kidney” and “CKD.” We then reviewed the abstracted data to remove any duplicates. An Apple device (iPhone X), an Android device (Xiaomi Redmi Note 9 Pro), and an Android emulator (Pixel 4 API 30) were used to complete the screening. The search was done within the following time period: 04/05/2021–07/08/2021. The numbers of apps that were initially identified and subsequently assessed are summarized in the PRISMA diagrams in Figs. 1 and 2.

We included apps in English that were designed specifically for SLE and LN patients. There were no restrictions on app cost. Apps were excluded if they had a target audience different from SLE/LN patients or lacked function. For apps that satisfied our inclusion criteria but had functional problems, attempts were made to contact the developer to ensure that we were not excluding any eligible apps. Other reasons for exclusion are detailed in Figs. 1 and 2.

Apps with vague functions were first discussed between AR and PD and later adjudicated by a third reviewer (CM) to determine inclusion. Conflicts were also resolved by the third reviewer. A Google Sheets spreadsheet was used to produce a final list of the included apps, with information about the developer, price, app version, and app content retrieved from each app.

The Mobile App Rating Scale (MARS), an objective and reliable technique for assessing app quality, was used to evaluate the included mHealth apps [13]. The MARS scale is categorized into five major areas of criteria, including four objective quality scales: engagement, functionality, aesthetics, and information, as well as one subjective quality scale, which were combined into a 23-item questionnaire. A 5-point scale (1, inadequate; 2, poor; 3, acceptable; 4, good; 5, excellent) is used to rate each of the 23 items. An option of “not applicable” was provided in instances where an item might not be applicable to all apps. Individual mean ratings of engagement, functionality, aesthetics, and information quality were combined to provide an overall mean app quality score. The subjective quality portion of the MARS is optional; therefore, it was excluded to ensure objectivity. In any circumstances where there was a disagreement (a difference of more than 2 points in any of the individual MARS subscale mean scores), a third reviewer was to be consulted (CM). However, the two reviewers had a high level of agreement, with ratings never deviating by more than the 2-point threshold. The reviewers also watched the MARS training video provided by the creators of the scale. If the reviewers needed clarification for any MARS elements, they first referred to the training video, and any lingering questions were discussed among the reviewers.

## Results

### Screening results for SLE apps

Of the 841 apps that were returned by our search, only 17 mHealth applications qualified for inclusion (Fig. 1). The common reasons for the exclusion of apps were duplicates, irrelevance to the topic of this study, and lack of function. Within the 17 apps ultimately included, nine were found exclusively in Google Play, and eight were found in both Google Play and App Store. Lupus (SLE) and Lupus Diary, priced at $3.99 and $4.99, respectively, needed to be purchased. Of note, our iOS screening for the search term “SLE” did not yield any additional hits. Of the 17 apps, fourteen provided educational information about the disease, four allowed for tracking patient-reported symptoms, and three included discussion forums. App characteristics are discussed in detail in Table 1.

**Table 1:** Systemic lupus erythematosus (SLE) app characteristics

App name	Function	Platform	Price	Developer	Affiliation
Home remedies for lupus	Educational	Android	Free	Hilltop_apps	Commercial
How to Cure Lupus	Educational	Android	Free	Apps How to Apps	Commercial
Lupie Diary	Maintain records, educational	iOS and Android	Free	Syamsi Dhuha Foundation	Commercial
Lupus (SLE)	Educational	iOS and Android	$3.99	Personal Remedies LLC	Commercial
Lupus Advice	Educational	Android	Free	moreFlow	Commercial
Lupus Corner Health Manager/LupusCorner Toolkit	Symptom tracking, discussion forum, educational	iOS and Android	Free	Progentec Diagnostics, Inc.	Commercial
Lupus Diary	Symptom tracking	iOS and Android	$4.99	cellHigh LLC	Commercial
Lupus Disease	Educational	Android	Free	Kalpesh Lakhani	Commercial
Lupus Ohio	Educational, support group, communication with healthcare provider	Android	Free	Lupus Foundation of America	Non-profit
Lupus Symptoms Treatment	Educational	Android	Free	Revolxa Inc.	Commercial
Lupus: Causes, Diagnosis, and Treatment	Educational	Android	Free	Health Info	Commercial
Lupus: Causes, Diagnosis, and Treatment	Educational	Android	Free	DevoDreamTeam	Commercial
LupusConnect Inspire Community	Support group, discussion forum	iOS and Android	Free	Inspire, Inc.	Commercial
LupusMinder	Symptom tracking	iOS and Android	Free	Hospital for Special Surgery	Non-profit
ManageMyLupus	Symptom tracking, educational	iOS and Android	Free	University of Alabama at Birmingham Medicine	Non-profit
MyLupusTeam/Lupus Support	Discussion forum, educational	iOS and Android	Free	MyHealthTeams	Commercial
Treating My Lupus	Educational	Android	Free	UAB Gout Aid	Non-profit

### MARS results

On a scale of 1–5, the mean (SD) MARS app quality score for all 17 apps included was 2.7 (± 0.4 SD) out of 5. The majority of the apps scored poorly on the basis of engagement and aesthetics but highly in functionality. As most apps only provided information about the disease with few opportunities for user engagement, the mean MARS engagement subscale score was 2.5 (± 0.5 SD). With most of the apps being straightforward and simple to use, apps scored high on the functionality aspect of the scale, receiving a MARS functionality subscale score of 3.5 (± 0.3 SD). The mean MARS aesthetics subscale score was 2.4 (± 0.6 SD) as most applications had poor visual designs, low-resolution images, and inconsistent styles. The vast majority of apps lacked credibility as the disease-related information provided on the app was not referenced or traceable to a credible information source or web page. Hence, they scored a mean MARS information subscale rating of 2.6 (± 0.5 SD). Table 2 details the individual and the overall mean MARS scores of each app.

**Table 2:** The Mobile App Rating Scale (MARS) assessment of SLE apps. The Mobile App Rating Scale runs from 1 to 5 for each category, with a score of 1 indicating poor quality and a score of 5 indicating high quality [13]. The subjective quality subscale was excluded from this study. The average of each subscale was used to obtain the overall score. The listed numbers are the means of the two independent scores from AR and PD

App name	MARS engagement	MARS functionality	MARS aesthetics	MARS information	Overall mean app quality score
Home Remedies for Lupus	2.2	3.25	1.5	2.2	2.288
How to Cure Lupus	1.8	2.75	1.65	2.4	2.15
Inspire/Lupus Connect Inspire Community	2.6	3.35	2.85	2	2.7
Lupie Diary	2.9	3.875	2.55	2.75	3.019
Lupus (SLE)	2.1	3.225	1.95	1.55	2.206
Lupus Advice	2.8	3.35	2.7	2.4	2.813
Lupus Corner Health Manager	2.9	3.25	2.75	2.95	2.963
Lupus Diary	3	3.375	1.5	2.1	2.494
Lupus Disease	2.8	3.55	2.65	2.875	2.969
Lupus Ohio	2.75	3.5	2.85	3	3.025
Lupus Symptoms Treatment	2.45	3	1.5	2.425	2.344
Lupus: Causes, Diagnosis, and Treatment	2	3.475	1.85	3	2.581
Lupus: Causes, Diagnosis, and Treatment	1.9	3.6	1.85	2	2.338
LupusMinder	2.9	3.5	3.1	2.35	2.963
ManageMyLupus	1.8	3.65	3.15	3.2	2.95
MyLupusTeam	2.8	3.6	2.85	2.35	2.9
Treating My Lupus	2.4	3.55	2.2	3	2.788
Mean	2.5	3.5	2.4	2.6	2.7
Standard deviation	0.5	0.3	0.6	0.5	0.4

### Highest scoring SLE apps

Lupus Ohio, Lupie Diary, and Lupus Corner Health Manager were the three highest scoring SLE applications. The Lupus Ohio app was developed by the Lupus Foundation of America and designed for members of the Greater Ohio Chapter, but the app can be used by any SLE patient. The app provides lupus-related information with a high level of credibility. Users of the app could also look for support groups and reach out to a health expert for any personal questions. Lupie Diary helps patients make and maintain their medical records. Lupus Corner Health Manager allows patients to track their symptoms and offered an interactive online community with discussion forums. All three applications offered adequate levels of user engagement, visual appeal, and content.

#### Lupus Ohio (overall MARS score 3.03)

Lupus Ohio is the official app of the Greater Ohio Chapter of the Lupus Foundation of America. This app provides patients with access to recent research articles and findings about lupus sourced from the website of the Lupus Foundation of America. As the research comes from a legitimate source, SLE patients are given credible information about the disease. The app allows patients to keep track of their medications and doctor visits. By reading the research updates, patients will have an idea of contemporary research and emerging therapies on the horizon. Users are given the opportunity to ask questions about their condition to a health educator through the organization’s website. As SLE can take an emotional toll on patients, healthcare providers may utilize this app by referring patients to the support groups offered by the organization. This app could be further optimized by including features for monitoring lupus-related symptoms and a medication reminder system to help with patient adherence to their treatments.

#### Lupie Diary (overall MARS score 3.02)

Lupie Diary app was developed to assist SLE patients store medical records, monitor medications, and record treatment plans received from their physicians. Since patients commonly access multiple care providers across different disciplines, and sometimes even across different hospitals, having ready access to one’s detailed medical history on the phone will quickly provide physicians with the patient’s comprehensive health status and medication history. Other functionalities include medication reminders and self-management tips sourced from credible sites. The app also provides basic information about SLE drawn from the Lupus Foundation of America. However, this app could be further optimized by offering recent news about lupus, doctor’s appointment reminders, and including features for lupus-related symptom tracking.

#### Lupus Corner Health Manager (overall MARS score 2.96)

By answering four questions on a 5-level scale, users can track their energy and general symptoms such as discomfort, fatigue, or pain. Users can also report side effects and the severity of their symptoms. The app also provides a scheduled medication reminder system. Users may also participate in an online discussion forum within the app, where they can seek support, ask questions, and get answers from other users through comments. The app also includes the latest news regarding SLE and alerts the user when new articles are published. This app could be further optimized by including features for communication with a healthcare expert in the field or patient-centered feedback based on the survey responses of users. Of note, none of the above apps allowed patients to monitor their renal disease status, either based on symptoms or based on point-of-care tests.

### Screening results for LN apps

Our search yielded 1152 apps, and only two were ultimately included (Fig. 2). The majority of the apps were excluded because they were not designed for LN patients and were irrelevant to the purpose of this study. Within the two apps that were included, Glomerulonephritis Disease was found solely in the Google Play Store and KidneyWell was found on both app stores. Of the two apps, Glomerulonephritis Disease provided educational content and KidneyWell offered telehealth services. Of note, KidneyWell is no longer available on both app stores. However, those that had downloaded the app prior to removal are still able to use it. App characteristics are further discussed in detail in Table 3.

**Table 3:** Lupus nephritis (LN) app characteristics

App name	Function	Platform	Price	Developer	Affiliation
Glomerulonephritis Disease	Educational	Android	Free	Bedieman	Commercial
KidneyWell^a^	Communication with health-care provider	iOS and Android	Free	Dallas Nephrology Associates	Commercial

^a^KidneyWell is no longer available on the Android and iOS platforms, but those who had already downloaded it can still use it

#### KidneyWell (overall MARS rating 2.76)

The KidneyWell app allows individuals diagnosed with kidney illness or disorders to meet virtually with renal specialists from a private setting, using a HIPAA-secure connection. The app offers credibility to patients as it was created the Dallas Nephrology Associates. Although this app is useful in a clinical setting by offering virtual appointments with a physician, the app does not provide any information about LN or allow for other types of patient engagement. Although this app is not designed specifically for patients with LN, we included it as LN patients could potentially use it for consultation purposes.

#### Glomerulonephritis Disease (overall MARS rating 2.28)

The Glomerulonephritis Disease app presented educational information about the condition. In addition to providing an overview of the disease, the app listed the common symptoms associated with LN and offered information about the diagnosis of the disease. The app also explained the differences between proliferative and non-proliferative glomerulonephritis. The information was not referenced to a legitimate source.

### MARS results

The mean (SD) MARS app quality score for the two apps included was 2.6 (± 0.4 SD) out of a maximum score of 5. The Glomerulonephritis Disease app scored the lowest in the sections of engagement and aesthetics. The KidneyWell app scored poorly in the engagement and information sections. The mean MARS engagement subscale score was 2.2 (± 0.5 SD) as opportunities for user engagement were very limited for both apps. Both apps scored high on the functionality section, receiving a MARS functionality subscale score of 3.5 (± 0.2 SD). The mean MARS aesthetics subscale score was 2.2 (± 0.7 SD), with KidneyWell offering a more pleasant visual appeal than Glomerulonephritis Disease. As Glomerulonephritis Disease came from a questionable source, KidneyWell had a higher information rating with both apps scoring a mean MARS information subscale rating of 2.4 (± 0.1 SD). Individual and overall mean MARS ratings for the two apps are shown in Table 4.

**Table 4:** The Mobile App Rating Scale (MARS) assessment of lupus nephritis (LN) apps

App name	MARS engagement	MARS functionality	MARS aesthetics	MARS information	Overall mean app quality score
Glomerulonephritis Disease	1.8	3.4	1.7	2.3	2.3
KidneyWell	2.5	3.7	2.6	2.4	2.8
Mean	2.2	3.5	2.2	2.4	2.6
Standard deviation	0.5	0.2	0.7	0.1	0.4

## Discussion

Our assessment of the current mHealth applications for SLE and LN patients indicates a substantial lack of patient-centered solutions for lupus patients with renal disease. The majority of the currently accessible mHealth applications for SLE and LN patients failed to adequately address the needs of the patients since they scored poorly on credibility, functionality, and/or interactivity.

Of the included apps for SLE, Lupus Ohio is the only one that offered a wide range of functionalities for SLE patients. As the app is designed by the Lupus Foundation of America, the organization already has a clear understanding of the needs of SLE patients and therefore delivered a relatively comprehensive app for these patients. The app provided credible educational material, communication with a health expert, and a medication and doctor visit tracker. On the other hand, it is only available on the Android app store and its target audience is specifically people from the Ohio chapter.

The bulk of the applications examined for LN, but ultimately excluded, only offered informational content about kidney diseases in general. Apps such as Kdigo, ASN Kidney News, The Nephrology Pocket, ResponsumforCKD, Nephrology Guide, and Kidney Diseases and Treatment appeared in our LN screening, but they did not satisfy the needs of LN patients as they failed to provide information specific to LN or offer any advanced functions. Of the 2 included apps, both Glomerulonephritis Disease and KidneyWell were rather limited in their functionalities offered to patients, as discussed under the “Results” section. The MARS scores for all of the apps included in this study had poor to mediocre ratings, demonstrating the modest quality of the available apps. The information domain scored the lowest, mainly because most of the available apps for SLE did not provide verifiable information. The majority of the apps were designed by third-party businesses with little relevance to renal disease. The applications scored poorly in the engagement and aesthetic arenas because developers did not focus on user engagement or the visual appeal of the apps. In order to produce more effective apps for SLE and LN patients, developers need to focus on offering a wider spectrum of functionalities desired by the patients. A combination of features such as support groups, discussion forums, communication with health experts, medication reminders, and regular symptom monitoring can be highly beneficial to patients. However, the apps reviewed fail to offer a comprehensive array of these features with the current apps only providing one function or the other. It would be important for app developers to continually optimize their apps based on patient feedback.

Mobile health apps, such as Lupie Diary, have the unique ability to document the nuances of the patient’s condition which a brief consultation with the provider may not capture. Apps such as these provide unparalleled opportunities for collecting PGHD/PRO from the patients in between visits, though none of the currently available apps is being used to accomplish this. Considering the patient can easily generate and maintain patient-generated health data (PGHD) and patient-reported outcomes (PROs) through such apps, these data have the potential to be integrated with commonly used electronic medical record (EMR) systems. If the PGHD/PRO collected through such apps were to be shared with the provider, it may allow the provider to remotely monitor patient symptoms and PROs and this could significantly influence patient management. With the burgeoning interest in digital phenotyping, such app features are likely to play an increasingly significant role in continuous patient monitoring in the coming years.

Despite the rise in mHealth technology as a supplemental approach for patients to self-manage their autoimmune diseases, the available applications created particularly for SLE and LN patients are inadequate. The existing instruments are of moderate quality, with limited functionality, and do not fully meet the rising needs of patients with SLE and LN. This review recommends the development of patient-centric mHealth technologies that offer wide-ranging functionalities and credible educational content. Also important is to empower the SLE/LN patient to self-monitor the disease from the comfort of home, particularly for early warning signs of renal involvement in SLE. Also required is an app-based screening of health-related quality of life (HRQoL) information, and apps that allow 2-way communication with the care provider. Likewise, it will become increasingly important to transition laboratory-based diagnostic tests into simple point-of-care test formats so that essential laboratory investigations can also be performed by the patient, from their home. These considerations become even more acutely warranted during a pandemic, such as in the world we currently live in.

## Conclusion

By facilitating the self-management of autoimmune illnesses, mobile health apps have the potential to empower individuals with renal disease. Though there is a growing interest in the development of mobile health technology for lupus patients, the available mHealth apps are of average quality, have limited functionality, and do not fully satisfy the rising demands of SLE and LN patients. The development of comprehensive, patient-centered mHealth apps is warranted to help patients manage the several challenges of lupus and improve their overall quality of life.

## Data Availability

All raw data generated in this work are freely available by contacting the authors.
